# Expression Profiles and Prognostic Value of Multiple Inhibitory Checkpoints in Head and Neck Lymphoepithelioma-Like Carcinoma

**DOI:** 10.3389/fimmu.2022.818411

**Published:** 2022-01-24

**Authors:** Wen-Qing Zou, Wei-Jie Luo, Yan-Fen Feng, Fang Liu, Shao-Bo Liang, Xue-Liang Fang, Ye-Lin Liang, Na Liu, Ya-Qin Wang, Yan-Ping Mao

**Affiliations:** ^1^ Department of Radiation Oncology, Sun Yat-sen University Cancer Center, State Key Laboratory of Oncology in South China, Collaborative Innovation Center of Cancer Medicine, Guangdong Key Laboratory of Nasopharyngeal Carcinoma Diagnosis and Therapy, Guangzhou, China; ^2^ Department of Medical Oncology, The Seventh Affiliated Hospital of Sun Yat-sen University, Shenzhen, China; ^3^ Department of Pathology, Sun Yat-sen University Cancer Center, State Key Laboratory of Oncology in South China, Collaborative Innovation Center of Cancer Medicine, Guangzhou, China; ^4^ Department of Pathology, The First People’s Hospital of Foshan, Foshan, China; ^5^ Department of Radiation Oncology, The Third Affiliated Hospital of Sun Yat-sen University, Guangzhou, China

**Keywords:** head and neck lymphoepithelioma-like carcinoma, multiple inhibitory checkpoints, tumor microenvironment, TNM stage, prognosis

## Abstract

**Background:**

Inhibitory checkpoints are promising antitumor targets and predictive biomarkers in a variety of cancers. We aimed to identify the expression levels and prognostic value of multiple inhibitory checkpoints supported by preclinical and clinical evidence in head and neck lymphoepithelioma-like carcinoma (HNLELC).

**Methods:**

The expression of seven inhibitory checkpoints were evaluated in the tumor nest (TN) and tumor stroma (TS) of 102 HNLELC specimens using immunohistochemistry and digital pathology, and an inhibitory checkpoint-based signature (ICS) was subsequently constructed using the LASSO Cox regression model.

**Results:**

PD-L1, B7H3, and IDO-1 were mostly expressed in the TN, with median H-score of TN vs TS: 63.6 vs 14.6; 8.1 vs 1.0; 61.5 vs 34.7 (all *P* < 0.001), whereas PD-1, TIM-3, LAG-3, and VISTA were mainly observed in the TS, with median H-score of TN vs TS: 0.2 vs 12.4, 3.4 vs 7.1, 6.2 vs 11.9, 16.4 vs 47.2 (all *P* < 0.001), respectively. The most common simultaneously expressed combinations consisted of PD-L1 + B7H3 + IDO-1 + TIM-3 + LAG-3 + VISTA and B7H3 + IDO-1 + TIM-3 + LAG-3 in the TN (both occurring in 8.8% of patients) and PD-L1 + B7H3 + IDO-1 in the TS (4.9%). In addition, high-ICS patients had shorter 5-year disease-free (40.6% vs 81.7%; *P* < 0.001), regional recurrence-free (63.5% vs 88.2%; *P* = 0.003), and overall survival (73.5% vs 92.9%; *P* = 0.006) than low-ICS patients. Multivariate analysis revealed that ICS represented an independent predictor, which could significantly complement the predictive performance of TNM stage for 3-year (AUC 0.724 vs 0.619, *P* = 0.014), 5-year (AUC 0.727 vs 0.640, *P* = 0.056), and 10-year disease-free survival (AUC 0.815 vs 0.709, *P* = 0.023).

**Conclusions:**

The expression of inhibitory checkpoints and ICS classifier may increase the prognostic value of the TNM staging system and guide the rational design of personalized inhibitory checkpoint blockade therapy in HNLELC.

## Introduction

Head and neck lymphoepithelioma-like carcinoma (HNLELC) is a rare malignant neoplasm histologically identical to nonkeratinizing undifferentiated nasopharyngeal carcinoma (NPC), but with origins other than the nasopharynx (1). Generally accepted treatment modalities for HNLELC consist of surgery and/or radiotherapy with or without adjuvant chemotherapy (2–4). Despite the favorable prognosis associated with HNLELC, over 30% of cases continue to experience recurrence, distant metastasis, and even death (2, 3, 5). Therefore, there is a need to identify valuable biomarkers that can provide therapeutic insight to further improve the outcome of HNLELC patients.

Emerging studies have demonstrated that inhibitory checkpoints expressed on the surface of tumor cells and/or immune cells negatively regulate the function of effector immune cells, which is an important mechanism of tumor immune escape (6). Antagonist antibodies that block inhibitory checkpoints have been devised to reverse immune resistance, representing the current frontier of cancer immunotherapy (7). To date, various inhibitory checkpoint blockades (e.g., anti-PD-1/PD-L1 and anti-LAG-3 antibodies) have been approved for the clinical application or subjected to preclinical/clinical studies (8). Of note, the results of several encouraging phase 2 trials and practice-changing randomized phase 3 trials exhibit unprecedented clinical efficacy of anti-PD-1 therapy in recurrent/metastatic NPC and other head and neck squamous cell carcinoma (HNSCC) (9, 10). HNLELC is characterized by substantial lymphocyte infiltration within the tumor microenvironment (2). Despite the presence of such infiltrates, the tumor remains capable of progressive growth, implying that the antitumor immunity of these lymphocytes may be suppressed. Therefore, inhibitory checkpoint blockades that can revitalize anergic T lymphocytes and alleviate immune suppression may represent an effective treatment strategy for HNLELC.

The expression status of inhibitory checkpoints is critical to guide inhibitory checkpoint blockade therapy. For example, high PD-L1 expression is significantly associated with a favorable clinical response to a PD-1/PD-L1 blockade in certain types of tumors, including HNSCC (10). In addition, a growing number of studies have demonstrated that inhibitory checkpoints present in the tumor microenvironment can predict prognosis in cancer patients (11, 12), acting as a complement to the anatomic-based TNM staging system (12). Thus, in the era of immunotherapy, it is essential to clarify the expression levels of inhibitory checkpoints to provide implications for immunotherapies targeting these checkpoints and identify those with prognostic value to improve the current TNM system ability of prognostic prediction in HNLELC.

Based on these considerations, we aimed to delineate the immunohistochemical expression of seven common inhibitory checkpoints in 102 HNLELC patients and develop an inhibitory checkpoint-based signature (ICS) to effectively predict the prognosis of HNLELC patients.

## Materials and Methods

### Study Specimens

This study retrospectively recruited paraffin-embedded tumor specimens from 102 HNLELC patients treated at Sun Yat-sen University Cancer Center (n = 78), the First People’s Hospital of Foshan (n = 21), and the Second People’s Hospital of Foshan (n=3) from 2001 to 2019. Inclusion criteria consisted of patients without distant metastasis and antitumor treatment prior to biopsy or surgery. Patients with incomplete clinicopathological characteristics and follow-up data were excluded. Tumor stages were reclassified according to the 8th edition of the American Joint Committee on Cancer (AJCC) TNM staging system (13). The included clinicopathological variables consisted of age, sex, smoking history, drinking history, pretreatment neutrophil-to-lymphocyte ratio (NLR), Epstein–Barr virus‐encoded RNA (EBER), T stage, N stage, and TNM stage. This study was approved by the institutional research ethical committees of all included institutions (B2020-375).

### Immunohistochemistry (IHC)

Slides stained with hematoxylin and eosin (H&E) were reviewed by two experienced pathologists (Dr Feng and Dr Liu) to reconfirm the pathological diagnosis and to select tumor tissue blocks containing tumor nest (TN) and tumor stroma (TS) for IHC staining as described previously (14). The following primary antibodies were used: anti-PD-L1 (clone E1L3N, 1:200; Cell Signaling Technology, CST, Beverly, Massachusetts, USA), anti-B7H3 (clone D9M2L, 1:400; CST), anti-IDO-1 (clone D5J4E, 1:800; CST), anti-PD-1 (clone D4W2J, 1:200; CST), anti-TIM-3 (clone D5D5R, 1:400; CST), anti-LAG-3 (clone D2G4O, 1:100; CST), and anti-VISTA (clone D1L2G, 1:200; CST). Human tonsil specimens with or without primary antibodies were used as positive or negative controls in each batch, respectively.

### Digital Image Analysis and Selection of Cut-Off Values

**Figure 1:**
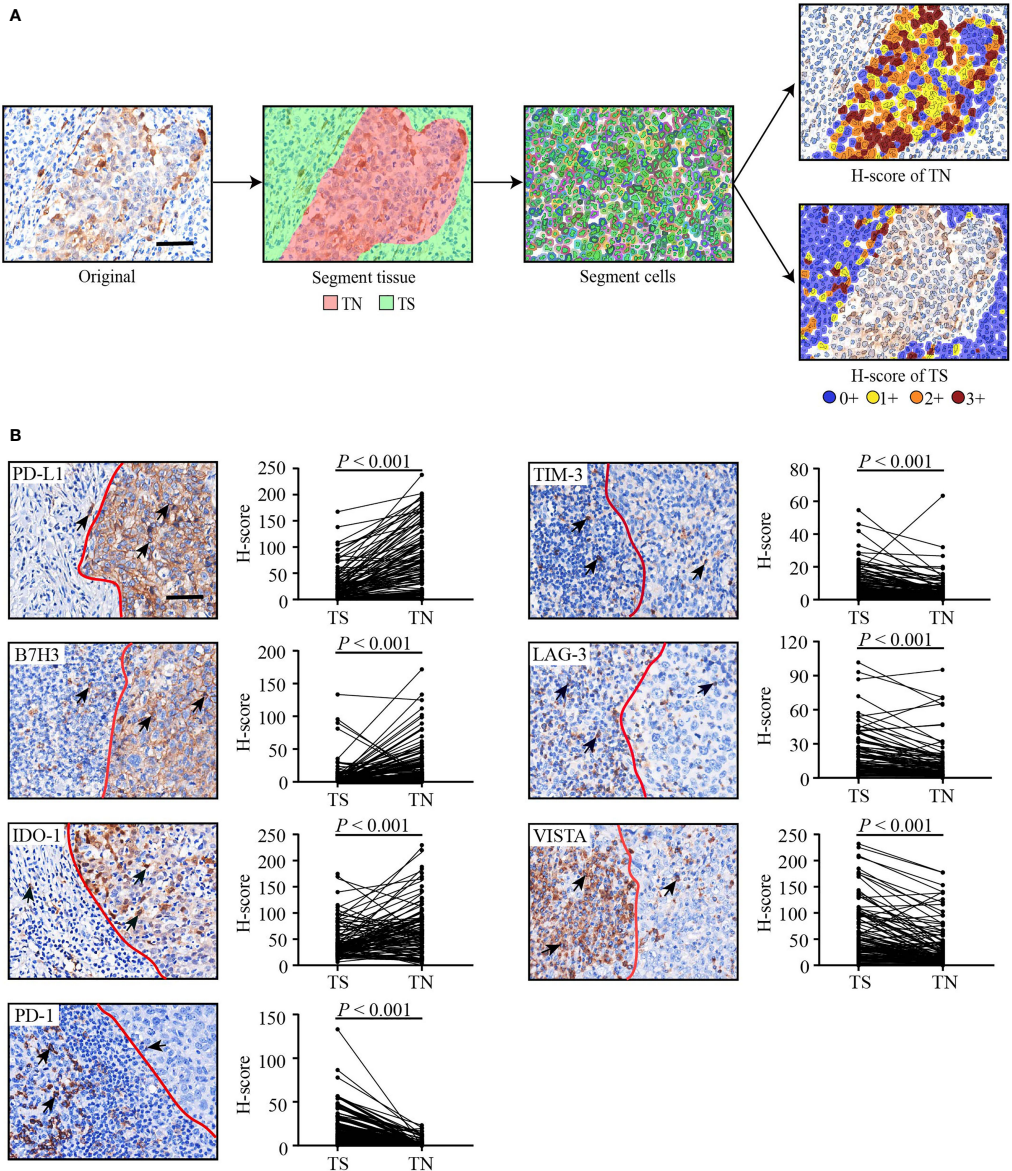
The flowchart of the digital image analysis and representative immunohistochemical images of seven inhibitory checkpoints. **(A)** The analysis flowchart. H-score = (% cells 3+) × 3 + (% cells 2+) × 2 + (% cells 1+). The staining intensity was graded as absent (0+), weak (1+), moderate (2+), or strong (3+). **(B)** Representative immunohistochemical images and H-scores of seven inhibitory checkpoints. Tumor tissues were divided into TN (right) and TS (left) by a red line. Positive cells (brown) are indicated by a black arrow. The scale bars represent 50 μm. Wilcoxon signed rank test for paired comparisons was used to calculate *P* values. TN, tumor nest; TS, tumor stroma.

IHC-stained slides were scanned at a high power (200×) (0.496 μm/pixel) using a Vectra 2.0.8 multispectral microscope system (Perkin Elmer, Waltham, Massachusetts, USA), and were subsequently quantified using inForm 2.1.1 software (Perkin Elmer). The flow of analysis was performed as follows (**Figure 1A**): 1) image preparation: the images were loaded and prepared for segmentation by converting them to optical density (OD); 2) segmenting tissue: tissue regions of interest were manually annotated as TN and TS; 3) segmenting cells: all individual cells and their associated cytoplasm within the segmented tissue categories were identified based on the nuclear signal and the distance to the nucleus using the cell segmentation tool; 4) scoring IHC: positive cells within each region were separately quantified as H-scores, which were calculated by the staining intensity (0, absent; 1+, weak; 2+, moderate; and 3+, strong) multiplied by the percentage (0 − 100) of positive cells at each intensity, which ranged from 0 to 300 (15). The parameters for cell segmentation and the thresholds of the different staining intensities were collectively confirmed by Dr Feng and Dr Liu who were blinded to the clinicopathological characteristics and outcomes of the patients. For each marker per patient, five representative fields were assessed and the average H-score was used as the final score.

The optimum cut-offs for the H-scores of 14 checkpoint features (seven checkpoints in the TN or TS) were selected based on their associations with disease-free survival (DFS) using X-tile software (version 3.6.1; Yale University, New Haven, CT, USA) (16). The patient distribution of each inhibitory checkpoint expression and the cut-off value of high and low expression are shown in **Supplementary Figure S1** and **Supplementary Table S1**. X-tile software is a popular bioinformatics tool for optimizing survival-based cutoff value by log-rank chi-square test. The optimal cut-off value was determined by the minimum *P* value with the highest χ^2^ value, and a corrected *P* value was calculated by Miller-Siegmund model (17).

### *In Situ* Hybridization (ISH) of EBER

EBV infection was assessed using an EBER ISH Kit (ZSGB-BIO, ISH-7001, Beijing, China) according to the manufacturer’s instructions. Known EBER-positive and EBER-negative sections were applied as positive and negative controls.

### ICS Construction

Due to the relatively small number of events compared with variables to be filtered, the least absolute shrinkage and selection operator (LASSO) Cox regression model was adopted to select the checkpoint features with a strong prognostic value and low correlation to prevent overfitting (18). The optimal value for λ was identified *via* 10-fold cross validation with 1-SE (standard error) criteria. Next, we constructed a multi-checkpoint feature-based classifier to predict the outcome of HNLELC patients. The ICS score was calculated as the sum of H-scores of the selected features as weighted by their regression coefficients. The LASSO Cox regression analysis was conducted using the R package “glmnet” (19).

### Statistical Analysis

Our primary endpoint was DFS, as defined as the time from the first day of treatment to any failures (local, reginal, or distant) or death from any causes. Secondary endpoints included regional recurrence-free survival (RRFS), time to first draining lymph node recurrence; and overall survival (OS), time to death.

Two groups were compared using a χ^2^ test or Fisher’s exact test for categorical variables and a Mann-Whitney U test and Wilcoxon signed rank test for nonpaired and paired continuous variables. Upset plot (https://www.omicstudio.cn/tool/43) was used to assess the co-expression of different inhibitory checkpoints (20, 21). A Pearson’s correlation test was used to calculate the correlation coefficients. The Kaplan-Meier method with a log-rank test was used to estimate DFS, RRFS, and OS. A univariate Cox regression analysis was used to generate the hazard ratio (HR) and 95% confidence interval (CI). Variables found to be significant in the univariate analysis (*P* ≤ 0.20) or of clinical significance were included in the multivariate analysis with the backward stepwise method to identify independent prognostic variables. Moreover, we established a combined predictive model using a multivariate Cox regression model. The β coefficient of each variable was divided by that of TNM stage and then rounded to an integer value to generated the risk score (12, 22). Next, we compared the predictive performance of the different models using a receiver operating characteristic (ROC) curve and time-dependent area of under the curve (AUC) analysis.

All statistical analyses with a two-sided test were conducted using IBM SPSS software (version 25; Property of IBM Corp., Armonk, NY, USA) and R software (version 4.0.2). A threshold of *P* < 0.05 was considered significant.

## Results

### Expression of Inhibitory Checkpoints

**Table 1** summarizes the baseline characteristics of 102 HNLELC patients from three independent institutions. Representative IHC images and the paired H-scores of seven inhibitory checkpoints in the TN and TS are presented in **Figure 1B**. The IHC-based image analysis revealed that the expression of PD-L1, B7H3, and IDO-1 were mainly detected in the TN, with median H-score of TN vs TS: 63.6 vs 14.6, 8.1 vs 1.0, 61.5 vs 34.7 (all *P* < 0.001), respectively. By contrast, PD-1, TIM-3, LAG-3, and VISTA were primarily expressed in the TS, with median H-score of TN vs TS: 0.2 vs 12.4, 3.4 vs 7.1, 6.2 vs 11.9, 16.4 vs 47.2 (all *P* < 0.001), respectively.

**Table 1:** Baseline Characteristics of 102 HNLELC Patients Stratified According to the ICS.

Characteristics	No. Of Patients (%) For ICS Stratification		P*
	Low Risk	High Risk	
**All**	81 (79.4)	21 (20.6)	
**Age, Years**			0.701
≤50	50 (61.7)	12 (57.1)	
>50	31 (38.3)	9 (42.9)	
**Sex**			0.642
Male	47 (58.0)	11 (52.4)	
Female	34 (42.0)	10 (47.61)	
**Smoking History**			0.890
Yes	12 (14.8)	4 (19.0)	
No	69 (85.2)	17 (81.0)	
**Drinking History**			1.000
Yes	10 (12.3)	2 (9.5)	
No	71 (87.7)	19 (90.5)	
**NLR**			1.000
≤4.5	70 (86.4)	18 (85.7)	
>4.5	11 (13.6)	3 (14.3)	
**EBER**			0.305
Positive	77 (95.1)	18 (85.7)	
Negative	4 (4.9)	3 (14.3)	
**Primary Site**			0.079
Salivary Gland	54 (66.7)	13 (61.9)	
Oropharynx	10 (12.3)	3 (14.3)	
Oral Cavity	5 (6.2)	1 (4.8)	
The Maxillary Sinus/ Nasal Cavity	3 (3.7)	4 (19.0)	
Else	9 (11.1)	0 (0.0)	
**T Stage^#^**			0.220
T1-T2	61 (75.3)	13 (61.9)	
T3-T4	20 (24.7)	8 (38.1)	
**N Stage^#^**			0.463
N0-N1	39 (48.1)	12 (57.1)	
N2-N3	42 (51.9)	9 (42.9)	
**TNM Stage^#^**			0.954
I-III	38 (46.9)	10 (47.6)	
IV	43 (53.1)	11 (52.4)	

*χ^2^ Test Or Fisher’s Exact Test.

^#^The 8th Edition Of The American Joint Committee On Cancer (AJCC) Classification.

HNLELC, Head And Neck Lymphoepithelioma-Like Carcinoma; ICS, Inhibitory Checkpoint-Based Signature; NLR, Neutrophil-To-Lymphocyte Ratio; EBER, Epstein-Barr Virus-Encoded Small RNA.

Due to a lack of a standard cut-off value for defining the expression of these checkpoints as either positive or negative in HNLELC, we described the distribution of patients using five H-score cut-offs (> 1, > 5, > 30, > 55, and > 100), which have been frequently used for the evaluation of PD-L1 (23–27). The median H-score of seven inhibitory checkpoints in the TN and TS was determined. As shown in **Table 2**, we observed high expression of PD-L1, IDO-1 and VISTA in both the TN and TS and PD-1 and LAG-3 in the TS, with a median H-score greater than 10. In contrast, the expression of B7-H3 in the TS and PD-1 in the TN were extremely low, with a median H-score less than or equal to 1.

**Table 2:** The Distribution of 102 HNLELC Patients for Each Cut-Off H-Score of Seven Inhibitory Checkpoints in the Tumor Nest And Tumor Stroma.

Markers	Median H-Score	No. Of Patients (%) For Each H-Score Cut-Off Value				
		> 1	> 5	> 30	> 55	> 100
**Tumor Nest**						
PD-L1	63.6	91 (89.2)	87 (85.3)	73 (71.6)	57 (55.9)	32 (31.4)
B7H3	8.1	75 (73.5)	61 (59.8)	23 (22.5)	12 (11.8)	5 (4.9)
IDO-1	61.5	101 (99.0)	100 (98.0)	79 (77.5)	58 (56.9)	18 (17.6)
PD-1	0.2	39 (38.2)	17 (16.7)	0 (0.0)	0 (0.0)	0 (0.0)
TIM-3	3.4	86 (84.3)	36 (35.3)	2 (2.0)	1 (1.0)	0 (0.0)
LAG-3	6.2	94 (92.2)	58 (56.9)	10 (9.8)	5 (4.9)	0 (0.0)
VISTA	16.4	100 (98.0)	81 (79.4)	36 (35.3)	23 (22.5)	10 (9.8)
**Tumor Stroma**						
PD-L1	14.6	90 (88.2)	74 (72.5)	31 (30.4)	14 (13.7)	4 (3.9)
B7H3	1.0	50 (49.0)	27 (26.5)	6 (5.9)	4 (3.9)	1 (1.0)
IDO-1	34.7	102 (100)	102 (100)	59 (57.8)	26 (25.5)	6 (5.9)
PD-1	12.4	92 (90.2)	80 (78.4)	23 (22.5)	7 (6.9)	1 (1.0)
TIM-3	7.1	98 (96.1)	70 (68.6)	4 (3.9)	0 (0.0)	0 (0.0)
LAG-3	11.9	97 (95.1)	78 (76.5)	22 (21.6)	5 (4.9)	1 (1.0)
VISTA	47.2	101 (99.0)	98 (96.1)	70 (68.6)	42 (41.2)	22 (21.6)

HNLELC, Head And Neck Lymphoepithelioma-Like Carcinoma.

### Correlation Among Inhibitory Checkpoints

**Figure 2:**
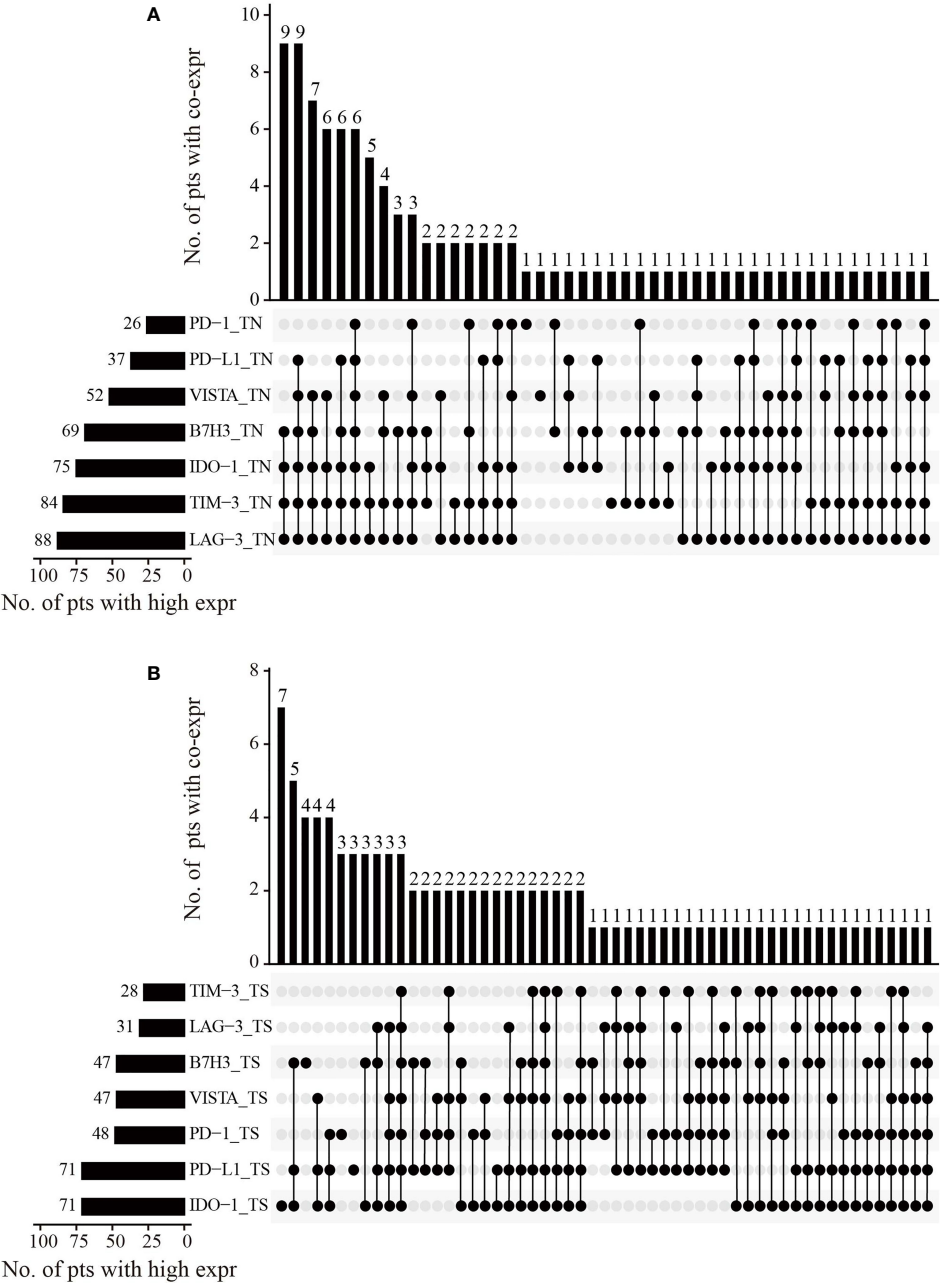
The co-expression combinations of seven inhibitory checkpoints in 102 HNLELC patients. Upset plots show the co-expression patterns of seven inhibitory checkpoints in the **(A)** TN and **(B)** TS. The horizontal bars on the left show the number of patients with high expressions for each checkpoint. The black beads on the bottom right indicate the high expression of corresponding checkpoints and the grey beads indicate the low expression of corresponding checkpoints. The black beads connected by the black lines represent the co-expression of the corresponding checkpoints with the status of high expression. The vertical bars on the top right show the number of patients with co-expression. HNLELC, head and neck lymphoepithelioma-like carcinoma; TN, tumor nest; TS, tumor stroma; pts, patients; expr, expression.

Based to the cut-off values of inhibitory checkpoints generated by X-tile software, patients were classified into high and low expression groups (**Supplementary Table S1**). Subsequently, we used Upset plots to assess the co-expression of the seven inhibitory checkpoints with the status of high expression in the TN and TS, respectively (**Figure 2**). In the TN, the most common co-expression combinations were PD-L1 + B7H3 + IDO-1 + TIM-3 + LAG-3 + VISTA and B7H3 + IDO-1 + TIM-3 + LAG-3, which were both observed in 8.8% of patients (9 of 102). In the TS, the most common co-expression combination was PD-L1 + B7H3 + IDO-1, occurring in 4.9% of patients (5 of 102).

Furthermore, in the bivariate correlation analysis for the expression of seven inhibitory checkpoints, the expression of most immune markers was significantly and positively correlated with each other, regardless in the TN or TS (r = 0.20 to 0.90; *P* = 0.042 to < 0.001). Strikingly, a significantly negative correlation was observed between B7H3_TS_ and PD-1_TS_ (r = -0.24; *P* = 0.017) (**Supplementary Figure S2**). Considering the efficacy of PD-1 and PD-L1 blockades for the treatment of a wide variety of cancers and the association between PD-L1 expression and the clinical response, we compared the expression status of other checkpoints in patients with low and high PD-L1 expression. Overall, patients with high PD-L1_TN_ expression also displayed higher levels of expression of six other inhibitory checkpoints compared with those with low PD-L1_TN_ expression, though the correlation with PD-1 and B7H3 expression did not reach statistical significance (**Supplementary Figure S3**).

### Association Between Inhibitory Checkpoint Expression and Clinicopathological Features

To explore the association between inhibitory checkpoint expression and patient characteristics, we analyzed differences in the expression of seven inhibitory checkpoints in patients with different clinical risk factors. As shown in **Supplementary Table S2**, several checkpoints were significantly associated with smoking history, EBV infection, T stage, and N stage, but not with drinking history or NLR. Patients with a history of smoking had a significantly higher B7H3_TN_ H-score (16.8 vs 6.5; *P* = 0.027) and lower PD-1_TS_ H-score (4.4 vs 13.1; *P* = 0.016) compared with those without a history of smoking. Moreover, high LAG-3 expression in the TN (7.0 vs 1.4; *P* = 0.006) and TS (13.6 vs 2.6; *P* = 0.003) was associated with EBV infection. In addition, T3-T4 stage patients expressed higher levels of PD-L1_TS_ compared to T1-T2 stage patients (36.3 vs 12.9; *P* = 0.012). N2-N3 stage patients expressed lower levels of VISTA_TN_ compared to N0-N1 stage patients (12.6 vs 22.0; *P* = 0.008).

### Development of an ICS Prognostic Classifier

**Figure 3:**
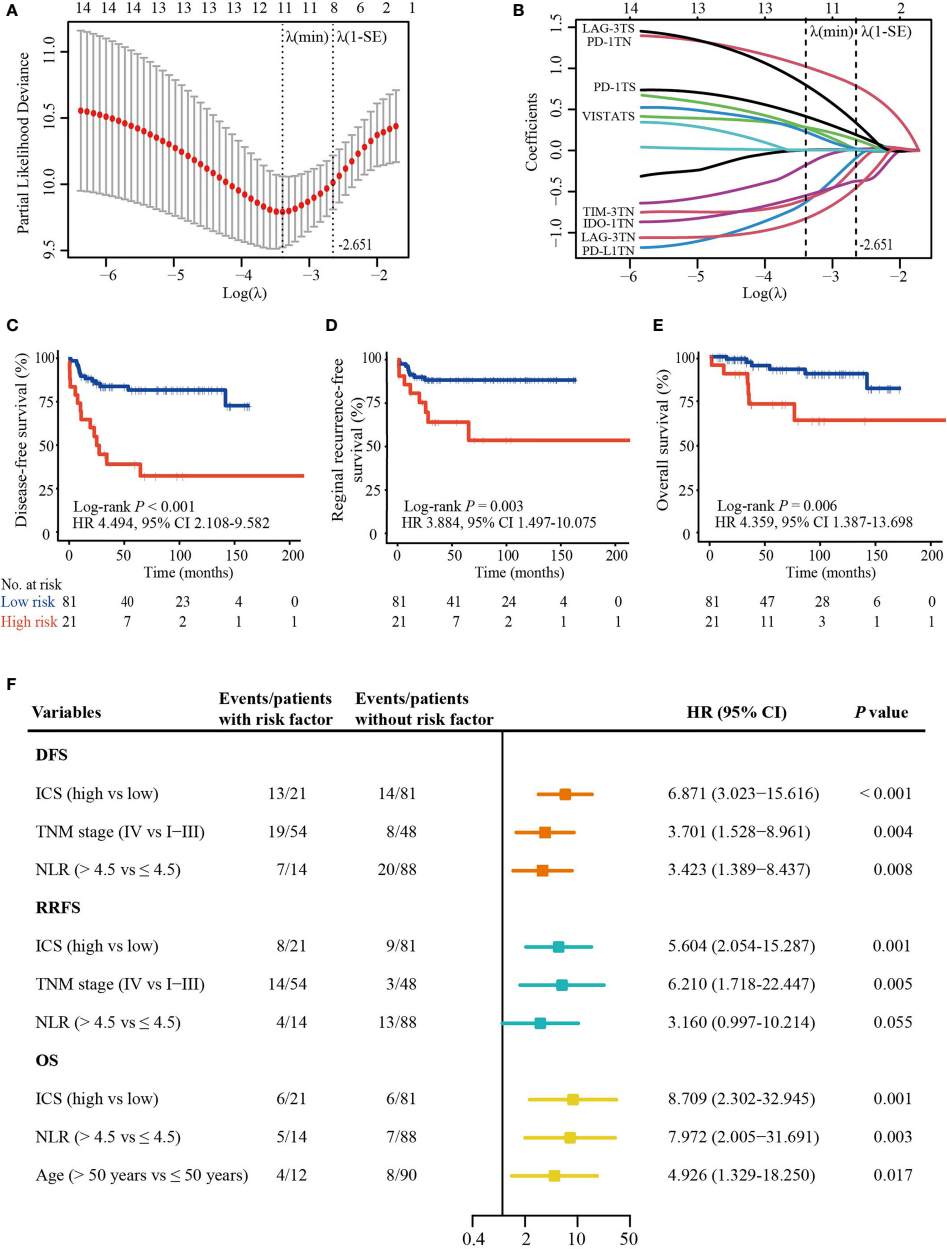
ICS construction and its prognostic value. **(A)** A ten-time cross validation for the selection of tuning parameter λ in the LASSO Cox regression model. The two dotted vertical line are drawn at the minimum partial likelihood deviance (min) and partial likelihood deviance ± 1 standard error (SE). Eight features with non-zero coefficients were selected based on the λ value of 0.071 with log(λ) = -2.651 using ten-time cross validation with the 1-SE criteria. **(B)** The coefficient profiles of the 14 checkpoint features included in the LASSO model. **(C–E)** The Kaplan-Meier curves of DFS, RRFS, and OS according to the ICS. **(F)** Plot shows significant variables in multivariate analysis. ICS, inhibitory checkpoint-based signature; LASSO, least absolute shrinkage and selection operator; NLR, neutrophil-to-lymphocyte ratio; DFS, disease-free survival; RRFS, reginal recurrence-free survival; OS, overall survival; HR, hazard ratio; CI, confidence interval; TN, tumor nest; TS, tumor stroma.

We investigated the prognostic impact of 14 checkpoint features for DFS (**Supplementary Figure S4**), RRFS (**Supplementary Figure S5**), and OS (**Supplementary Figure S6**). The integrated analysis of multiple biomarkers can provide better prognostic efficiency (28). Based on λ = 0.071 with log (λ) = -2.651 in the LASSO Cox regression model, an optimal subset of checkpoint features associated with DFS was selected, including PD-L1_TN_, IDO-1_TN_, PD-1_TN_, PD-1_TS_, TIM-3_TN_, VISTA_TS_, LAG-3_TN_, and LAG-3_TS_ (**Figures 3A, B**). The following formula was used: ICS score = (-0.06292027 × H-score of PD-L1_TN_) + (-0.40215406 × H-score of IDO-1_TN_) + (0.77959922 × H-score of PD-1_TN_) + (0.22403960 × H-score of PD-1_TS_) - (0.17722631× H- score of TIM-3_TN_) + (0.31940380 × H-score of LAG-3_TS_) - (0.45552439 × H-score of LAG-3_TN_) + (0.16116521 × H-score of VISTA_TS_).

Using the X-tile tool (**Supplementary Figure S7**), 81 (79.4%) patients were classified as low-risk (ICS score ≤ 4.2) and 21 (20.6%) as high-risk (ICS score > 4.2). Clinicopathological characteristics were equally distributed in two groups (**Table 1**). High-ICS patients experienced a shorter 5-year DFS (40.6% vs. 81.7%; HR 4.494; 95% CI, 2.108 – 9.582; *P* < 0.001), RRFS (63.5% vs. 88.2%; HR 3.884; 95% CI, 1.497 – 10.075; *P* = 0.003) and OS (73.5% vs. 92.9%; HR 4.359; 95% CI, 1.387 – 13.698; *P* = 0.006) than low-ICS patients (**Figures 3C–E**). Furthermore, following the univariable analysis (**Supplementary Table S3**), multivariate analysis revealed that ICS was an independent predictor of DFS (HR 6.871; 95% CI, 3.023 − 15.616; *P* < 0.001), RRFS (HR 5.604; 95% CI, 2.054 − 15.287; *P* = 0.001) and OS (HR 8.709; 95% CI, 2.302 − 32.945; *P* = 0.001) (**Figure 3F**).

### Development of a Combined Predictive Model

The purely anatomic-based TNM stage provides limited predictive performance for cancer prognosis, implying that it needs additional prognostic factors to improve its prognostic ability. We did a multivariate Cox regression analysis to develop a combined model to predict DFS risk based on its independent predictors: ICS, TNM stage, and NLR (**Figure 3F**). The risk score of each independent predictor is shown in **Supplementary Table S4**. ROC analysis found that the AUCs of ICS alone (AUC 0.687 vs 0.619, *P* = 0.340) and NLR alone (AUC 0.583 vs 0.619, *P* = 0.585) were equal to TNM stage alone (**Supplementary Figure S8**).

**Figure 4:**
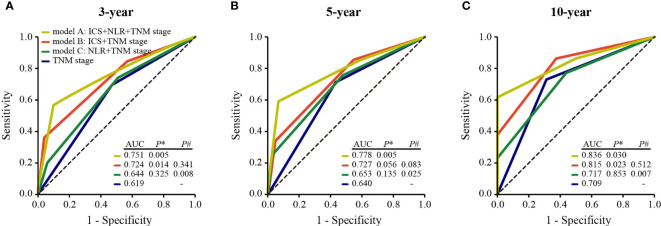
Comparisons of the predictive ability of model A, model B, model C, and TNM stage alone for 3-year **(A)** 5-year **(B)**, and 10-year DFS **(C)**. P* values show the comparisons of the AUC of model A, model B, or model C with that of TNM stage. *P#* values show the comparisons of the AUC of model B or model C with that of model A. ICS, inhibitory checkpoint-based signature; NLR, neutrophil-to-lymphocyte ratio; DFS, disease-free survival; AUC, the area of under the receiver operating characteristic curve.

To develop a simple model with a good predictive ability, we established three models: model A, TNM stage combining with ICS and NLR; model B, TNM stage combining with ICS; model C, TNM stage combining with NLR. Time-dependent ROC analysis was used to compare the predictive sensitivity and specificity of three models for 3-year, 5-year, and 10-year DFS. Among the three models, these was no significant difference in predictive ability between model A and model B for 3-year (AUC 0.751 vs 0.724, *P#* = 0.341), 5-year (AUC 0.778 vs 0.727, *P#* = 0.083), and 10-year DFS (AUC 0.836 vs 0.815, *P#* = 0.512). Whereas the predictive performance of model A was significantly superior to that of model C for 3-year (AUC 0.751 vs 0.644, *P#* = 0.008), 5-year (AUC 0.778 vs 0.653, *P#* = 0.025), and 10-year DFS (AUC 0.836 vs 0.717, *P#* = 0.007) (**Figures 4A–C**). In addition, model B displayed better predictive ability than TNM stage alone for 3-year (AUC 0.724 vs 0.619, P* = 0.014), 5-year (AUC 0.727 vs 0.640, P* = 0.056), and 10-year DFS (AUC 0.815 vs 0.709, P* = 0.023). Whereas there was no significant difference in the AUCs of 3-year (AUC 0.644 vs 0.619, P* = 0.325), 5-year (AUC 0.653 vs 0.640, P* = 0.135), and 10-year DFS (AUC 0.717 vs 0.709, P* = 0.853) between model C and TNM stage alone (**Figures 4A–C**).

## Discussion

Inhibitory checkpoint blockades have been proven to be effective antitumor strategies in multiple types of cancer (9, 10, 29). In this study, we investigated the abundance and co-expression patterns of seven inhibitory checkpoints as well as clinicopathological factors that may affect the expression of inhibitory checkpoints, which provided a basis for inhibitory checkpoint blockade therapy in HNLELC patients. In order to accurately identify patients with high risk who deserve intensified treatment, such as addition of immunotherapy, we developed an eight-feature-based ICS prognostic classifier that substantially improved the predictive ability for DFS when combined with the TNM stage.

Over the past few decades, inhibitory checkpoint blockades, represented by anti-PD-1/PD-L1 antibodies, have achieved unparalleled clinical success in multiple cancer types (30). In addition to PD-1/PD-L1, substantial evidence suggests that a variety of other inhibitory checkpoint molecules might exist in the tumor microenvironment [e.g., B7H3 (31), IDO-1 (32), LAG-3 (33), TIM-3 (34), and VISTA (35)], which also participate in the regulation of tumor immune escape. Thus, we investigated the expression of seven inhibitory checkpoints supported by preclinical and clinical evidence, which might provide a fundamental basis for future individualized immunotherapy targeting these checkpoints in HNLELC. In particular, abundant PD-L1 expression was observed in HNLELC patients. Given that response to a PD-1 blockade is more frequently reported in patients with high PD-L1 expression (36), a PD-1 blockade may be a potential candidate for the treatment of HNLELC.

Unfortunately, anti-PD-1/PD-L1 monotherapy has had a benefit on the long-term survival for only a fraction of cancer patients. One possible explanation is that the simultaneous existence of other inhibitory checkpoints might aggravate lymphocyte exhaustion and dysfunction, blocking the PD-1/PD-L1 signaling pathway alone is insufficient to restore immune function. Recent studies have demonstrated that combination therapies targeting two or more inhibitory checkpoints (e.g., PD-1 and LAG-3) (33), can achieve synergistic antitumor efficacy through nonredundant pathways to activate T cells. Therefore, understanding the co-expression and correlation of various inhibitory checkpoints should be highlighted to design more effective combination immunotherapy. The findings of this study indicate that the most common simultaneous expression patterns consisted of PD-L1 + B7H3 + IDO-1 + TIM-3 + LAG-3 + VISTA and B7H3 + IDO-1 + TIM-3 + LAG-3 in the TN and PD-L1 + B7H3 + IDO-1 in the TS. Moreover, PD-L1 expression was positively correlated with the expression of several other inhibitory checkpoints, suggesting the potential simultaneous involvement of multiple inhibitory checkpoint pathways in the HNLELC immune evasion mechanisms. Thus, the combination of several inhibitory checkpoint blockades (e.g., anti-PD-1 or anti-PD-L1 with other inhibitory checkpoint blockades) may improve clinical efficacy of HNLELC patients.

An accurate prognostic evaluation is critical for the selection of appropriate treatment. The TNM staging system is currently the most important benchmark for defining prognosis and to determine the treatment strategies for HNLELC (13). However, for many cancer patients, the purely anatomic-based staging provides only partial insight, which is useful but insufficient. Recent studies have revealed that the expression of inhibitory checkpoints is not only associated with immunotherapeutic benefits (37), but can also predict the prognosis of cancer patients (12). Integrating multiple biomarkers into a single model would increase the prognostic value (28). The present study constructed an ICS classifier based on the eight features containing critical prognostic information in the LASSO model, which provided a prognostic prediction independent of TNM stage. Furthermore, compared with other clinicopathological variables, the ICS classifier was a better variable that significantly complemented the prognostic performance of TNM stage. These results indicate the importance of the host immune status for predicting the prognosis of HNLELC patients, which may function as a complement to the TNM staging system.

Multiple studies have suggested that chronic infection and cancer often induce T cell exhaustion and the upregulation of multiple inhibitory checkpoints (38, 39). HNLELC has been reported to be tightly associated with EBV in regions with a high incidence (3, 40). In the present study, 95 of 102 cases were EBER-positive, and these patients displayed high LAG-3 expression, in line with the findings in a previous study of gastric cancer (41). Furthermore, we observed high PD-L1_TS_ expression in T3-T4 stage patients and low VISTA_TN_ expression in N2-N3 stage patients, suggesting that these markers are probably associated with tumor progression. It known that some lifestyle factors, such as tobacco smoking and alcohol drinking, are significant risk factors for head and neck cancer (42) and are closely associated with its prognosis (43). In order to explore whether smoking and drinking history affects immune microenvironment, we investigated the correlations of inhibitory checkpoint expression with smoking and drinking and found that patients with smoking history had significantly higher B7H3_TN_ and lower PD-1_TS_, suggesting that smoking may affect the expression of B7H3 and PD-1, which was consistent with previous studies (44, 45). Although the exact mechanism of how smoking affect the expression of B7H3 and PD-1 is unknown, studies have demonstrated that smoking is characterized by a high frequency of somatic mutation burdens in multiple cancers including head and neck squamous cell carcinomas (46), which may contribute to the improved response to immunotherapy (47, 48). Thus, the mechanism of the association between smoking and inhibitory checkpoints deserves to be further explored.

There were several limitations in this study that are important to note. First, since this was a retrospective study with a small sample size, external validation is required. However, given the low incidence of HNLELC, the present study represents a relatively large cohort. Second, previous studies have suggested that the load of EBV-DNA in the peripheral blood contains more precise information than the EBER status of the tumor tissue (49). However, due to the limited technological background, data pertaining to the EBV-DNA load in HNLELC was not available. Third, we did not investigate inhibitory checkpoint expression on different immune cell subpopulations and the colocalization of different inhibitory checkpoints using multiple immunohistochemistry staining. Fourth, we only focused on seven inhibitory checkpoints, other important immune checkpoints, such as stimulatory checkpoints (e.g., ICOS and OX40) (50, 51), may have been neglected. Clearly, much remains to be done to gain a better understanding of the HNLELC immune microenvironment.

In conclusion, this study demonstrated the heterogeneous distribution of seven inhibitory checkpoints in the TN and TS and developed an associated ICS classifier in HNLELC, which added the prognostic value to the TNM staging system. Together, our findings facilitate a more accurate patient prognostic stratification and pave the way for the development of immunotherapy targeting these checkpoints in HNLELC.

## Data Availability Statement

The raw data supporting the conclusions of this article has been uploaded the Research Data Deposit platform (https://www.researchdata.org.cn) (RDDA2022568951).

## Ethics Statement

The studies involving human participants were reviewed and approved by Sun Yat-sen University Cancer Center. Written informed consent to participate in this study was excused by the institutional review board.

## Author Contributions

Conception and design: Y-PM, Y-QW, and NL. Development of methodology: Y-FF, FL, W-QZ, and Y-QW. Collection of data: W-QZ, W-JL, X-LF, and S-BL. Data analysis and interpretation: W-QZ, W-JL, and Y-LL. Writing and revision of the manuscript: Y-PM, W-QZ, Y-QW, NL, W-JL, X-LF, S-BL, and Y-LL. All authors read and approved the final manuscript.

## Funding

This study was supported by grants from the National Natural Science Foundation of China (82102875, 81672988).

## Conflict of Interest

The authors declare that the research was conducted in the absence of any commercial or financial relationships that could be construed as a potential conflict of interest.

## Publisher’s Note

All claims expressed in this article are solely those of the authors and do not necessarily represent those of their affiliated organizations, or those of the publisher, the editors and the reviewers. Any product that may be evaluated in this article, or claim that may be made by its manufacturer, is not guaranteed or endorsed by the publisher.

## Abbreviations

HNLELC, head and neck lymphoepithelioma-like carcinoma; ICS, inhibitory checkpoint-based signature; TN, tumor nest; TS, tumor stroma; LASSO, least absolute shrinkage and selection operator; ROC, receiver operating characteristic; AUC, the area under the ROC curve; DFS, disease-free survival; RRFS, reginal recurrence-free survival; OS, overall survival; AJCC, American Joint Committee on Cancer; IHC, immunochemistry; H&E, hematoxylin and eosin; NLR, neutrophil-to-lymphocyte ratio; EBV, Epstein–Barr virus; EBER, Epstein–Barr virus-encoded small RNA; HR, hazard ratio; CI, confidence interval; SE, standard error.
