# Supplementary material for: Expression Profiles and Prognostic Value of Multiple Inhibitory Checkpoints in Head and Neck Lymphoepithelioma-Like Carcinoma
Source: Front Immunol. 2022 Jan 24;13:818411. doi: 10.3389/fimmu.2022.818411 (PMC8818848; doi:10.3389/fimmu.2022.818411)
Supplement: Supplementary file 1 [file DataSheet_1.docx]

Supplementary Material

**Supplementary Figure Legends**

Figure S1. The distribution of patients with high and low expression of seven inhibitory checkpoints in the TN and TS based on the optimal cutoff H-score using X-tile software. The values of X axis are the minimum H-score, the optimal cutoff H-score, and maximum H-score, respectively. The blue represents low expression and the grey indicates high expression. TN, tumor nest; TS, tumor stroma.

Figure S2. The correlation heatmap of seven inhibitory checkpoints in the TN and TS. The color of the heatmap represents the strength of the correlation. Red represents a positive correlation, whereas blue represents a negative correlation. * *P* < 0.05; ** *P* < 001; *** *P* < 0.001. *P* value was calculated by a Pearson’s correlation test. TN, tumor nest; TS, tumor stroma.

Figure S3. The expression of other six inhibitory checkpoints in patients with a high and low PD-L1_TN_ H-score. The median H-score of each marker is shown above the bar. *P* value was calculated by Mann-Whitney U test. TN, tumor nest; TS, tumor stroma.

Figure S4. Kaplan-Meier curves of disease-free survival according to 14 checkpoint features in HNLELC patients. TN, tumor nest, TS, tumor stroma; HR, hazard ratio; CI, confidence interval.

Figure S5. Kaplan-Meier curves of reginal recurrence-free survival based on 14 checkpoint features. TN, tumor nest; TS, tumor stroma; HR, hazard ratio; CI, confidence interval.

Figure S6. Kaplan-Meier curves of overall survival based on 14 checkpoint features. TN, tumor nest; TS, tumor stroma; HR, hazard ratio; CI, confidence interval.

Figure S7. The X-tile plots determining the optimal cut-off value of high and low risk patients according to ICS score. (A) Coloration of the plot represents the strength of association. Red represents a negative association between ICS and disease-free survival, whereas green indicates a positive association. The optimal cut-off point is the position of the black dot on the x-axis. (B) The ICS histogram presents an optimal cut-off value of 4.2. (C) Kaplan-Meier curves for ICS according to the generated cut-off value. ICS, inhibitory checkpoint-based signature.

Figure S8. The comparisons of predictive accuracy of ICS alone or NLR alone with that of TNM stage alone for DFS. *P* values show the comparisons of the AUC of ICS alone or NLR alone with that of TNM stage alone. ICS, inhibitory checkpoint-based signature; NLR, neutrophil-to-lymphocyte ratio; DFS, disease-free survival; AUC, the area of under the receiver operating characteristic curve.

Supplementary Tables

**Table S1. The DFS-based cut-off H-score for low and high expression of inhibitory checkpoints generated by X-tile software.**

| **Markers** | **Cut-off**  **H-score** | **No. of patients**  **(Low/High group)** | **Events**  **(Low/High group)** |
| --- | --- | --- | --- |
| Total |  | 102 | 27 |
| Tumor nest |  |  |  |
| PD-L1 | 84.8 | 65/37 | 20/7 |
| B7H3 | 2.4 | 33/69 | 11/16 |
| IDO-1 | 33.2 | 27/75 | 13/14 |
| PD-1 | 2.6 | 76/26 | 13/14 |
| TIM-3 | 1.2 | 18/84 | 7/20 |
| LAG-3 | 1.6 | 14/88 | 7/20 |
| VISTA | 15.0 | 50/52 | 10/17 |
| Tumor stroma |  |  |  |
| PD-L1 | 5.4 | 31/71 | 5/22 |
| B7H3 | 1.4 | 55/47 | 17/10 |
| IDO-1 | 24.4 | 31/71 | 11/16 |
| PD-1 | 12.6 | 54/48 | 8/19 |
| TIM-3 | 14.4 | 74/28 | 18/9 |
| LAG-3 | 22.8 | 71/31 | 15/12 |
| VISTA | 49.7 | 55/47 | 10/17 |

DFS, disease-free survival.

**Table S2. Correlation between inhibitory checkpoint expression (median H-score) and clinicopathological characteristics in 102 HNLELC patients.**

| **Marker** | **Smoking history** | |  | **Drinking history** | |  | **NLR** | |  | **EBER** | |  | **T stage** | |  | **N stage** | |  |
| --- | --- | --- | --- | --- | --- | --- | --- | --- | --- | --- | --- | --- | --- | --- | --- | --- | --- | --- |
|  | **No** | **Yes** | ***P**** | **No** | **Yes** | ***P**** | **≤ 4.5** | **> 4.5** | ***P**** | **Negative** | **Positive** | ***P**** | **T1-T2** | **T3-T4** | ***P**** | **N0-N1** | **N2-N3** | ***P**** |
| **Tumor nest** |  |  |  |  |  |  |  |  |  |  |  |  |  |  |  |  |  |  |
| PD-L1 | 67.7 | 54.8 | 0.682 | 71.3 | 46.9 | 0.377 | 69.6 | 63.6 | 0.576 | 72.0 | 62.4 | 0.508 | 57.0 | 79.1 | 0.188 | 72.4 | 49.4 | 0.114 |
| B7H3 | 6.5 | 16.8 | **0.027** | 7.4 | 14.4 | 0.095 | 7.7 | 9.8 | 0.303 | 5.8 | 8.2 | 0.666 | 6.4 | 11.0 | 0.200 | 5.6 | 12.4 | 0.121 |
| IDO-1 | 61.7 | 59.0 | 0.402 | 61.5 | 62.3 | 0.926 | 61.5 | 65.5 | 0.838 | 11.0 | 61.6 | 0.149 | 63.2 | 49.1 | 0.097 | 59.0 | 61.6 | 0.774 |
| PD-1 | 0.2 | 0.0 | 0.378 | 0.2 | 0.0 | 0.525 | 0.2 | 0.1 | 0.645 | 0.0 | 0.2 | 0.899 | 0.0 | 0.5 | 0.371 | 0.0 | 0.2 | 0.817 |
| TIM-3 | 3.5 | 2.3 | 0.945 | 3.6 | 2.3 | 0.421 | 3.5 | 3.1 | 0.573 | 1.4 | 3.6 | 0.166 | 3.3 | 3.6 | 0.546 | 3.4 | 3.2 | 0.666 |
| LAG-3 | 6.9 | 5.0 | 0.431 | 7.3 | 4.6 | 0.189 | 6.0 | 9.5 | 0.569 | 1.4 | 7.0 | **0.006** | 6.1 | 8.5 | 0.462 | 6.0 | 7.0 | 0.761 |
| VISTA | 18.4 | 9.9 | 0.341 | 18.4 | 6.2 | 0.187 | 15.9 | 17.0 | 0.981 | 22.0 | 16.0 | 0.807 | 15.9 | 17.2 | 0.741 | 22.0 | 12.6 | **0.008** |
| **Tumor stroma** |  |  |  |  |  |  |  |  |  |  |  |  |  |  |  |  |  |  |
| PD-L1 | 15.2 | 13.8 | 0.930 | 16.7 | 11.1 | 0.589 | 13.6 | 19.5 | 0.255 | 6.2 | 14.6 | 0.258 | 12.9 | 36.3 | **0.012** | 17.6 | 13.2 | 0.464 |
| B7H3 | 0.7 | 3.4 | 0.112 | 0.7 | 7.1 | 0.059 | 0.7 | 3.3 | 0.195 | 0.4 | 1.0 | 0.824 | 0.6 | 3.8 | 0.140 | 0.6 | 1.0 | 0.702 |
| IDO-1 | 35.1 | 33.8 | 0.825 | 34.2 | 35.1 | 0.682 | 32.5 | 44.3 | 0.180 | 31.8 | 35.2 | 0.634 | 32.3 | 35.8 | 0.534 | 31.8 | 35.6 | 0.356 |
| PD-1 | 13.1 | 4.4 | **0.016** | 12.7 | 4.9 | 0.062 | 12.5 | 8.8 | 0.439 | 19.8 | 12.4 | 0.439 | 12.6 | 11.6 | 0.719 | 13.2 | 10.4 | 0.301 |
| TIM-3 | 7.1 | 6.8 | 0.720 | 7.4 | 5.3 | 0.347 | 7.6 | 6.9 | 0.719 | 6.4 | 7.2 | 0.746 | 6.5 | 11.9 | 0.055 | 8.6 | 5.8 | 0.430 |
| LAG-3 | 13.2 | 9.7 | 0.288 | 13.2 | 8.0 | 0.184 | 11.6 | 20.9 | 0.280 | 2.6 | 13.6 | **0.003** | 11.8 | 14.7 | 0.160 | 11.4 | 13.6 | 0.971 |
| VISTA | 48.3 | 43.3 | 0.949 | 48.3 | 43.3 | 0.663 | 41.6 | 72.0 | 0.149 | 26.2 | 48.6 | 0.252 | 39.5 | 63.2 | 0.112 | 43.6 | 48.6 | 0.960 |

*Mann-Whitney U test.

HNLELC, head and neck lymphoepithelioma-like carcinoma; NLR, neutrophil-to-lymphocyte ratio; EBER, Epstein–Barr virus-encoded small RNA.

**Table S3. Univariate Cox regression analysis of clinicopathological characteristics and survival in 102 HNLELC patients.**

| Variables | DFS | |  | RRFS | |  | OS | |
| --- | --- | --- | --- | --- | --- | --- | --- | --- |
|  | HR (95% CI) | *P** |  | HR (95% CI) | *P** |  | HR (95% CI) | *P** |
| ICS (high vs low) | 4.494 (2.108-9.582) | **<0.001** |  | 3.884 (1.497-10.075) | **0.005** |  | 4.359 (1.387-13.698) | **0.012** |
| Age (> 50 years vs ≤ 50 years) | 1.370 (0.644-2.916) | 0.414 |  | 1.381 (0.532-3.581) | 0.507 |  | 3.416 (1.025-11.380) | **0.045** |
| Sex (male vs female) | 1.328 (0.608-2.901) | 0.477 |  | 1.141 (0.434-2.998) | 0.789 |  | 4.273 (0.933-19.566) | 0.061 |
| Smoking history (yes vs no) | 1.196 (0.451-3.172) | 0.720 |  | 0.676 (0.155-2.957) | 0.603 |  | 3.171 (0.927-10.849) | 0.066 |
| Drinking history (yes vs no) | 1.220 (0.420-3.542) | 0.715 |  | 0.430 (0.057-3.242) | 0.413 |  | 4.009 (1.173-13.703) | **0.027** |
| NLR (> 4.5 vs ≤ 4.5) | 2.638 (1.111-6.263) | **0.028** |  | 2.497 (0.811-7.685) | 0.111 |  | 4.753 (1.505-15.014) | **0.008** |
| EBER (positive vs negative) | 0.378 (0.130-1.099) | 0.074 |  | 0.340 (0.097-1.184) | 0.090 |  | 0.927 (0.120-7.194) | 0.942 |
| T stage (T3-T4 vs T1-T2) | 1.561 (0.714-3.410) | 0.264 |  | 1.060 (0.373-3.011) | 0.912 |  | 1.767 (0.560-5.574) | 0.332 |
| N stage (N2-N3 vs N0-N1) | 1.830 (0.835-4.009) | 0.131 |  | 3.517 (1.146-10.793) | **0.028** |  | 2.250 (0.673-7.521) | 0.188 |
| TNM Stage (IV vs I-III) | 2.446 (1.061-5.639) | **0.036** |  | 4.570 (1.312-15.922) | **0.017** |  | 2.166 (0.640-7.329) | 0.214 |

*Univariate Cox proportional hazards regression model.

HNLELC, head and neck lymphoepithelioma-like carcinoma; ICS, inhibitory checkpoint-based signature; NLR, neutrophil-to-lymphocyte ratio; EBER, Epstein-Barr virus-encoded small RNA; DFS, disease-free survival; RRFS, regional recurrence-free survival; OS, overall survival; HR, hazard ratio; CI, confidence interval.

**Table S4. Risk score based on β coefficient and hazard ratio (HR) in multivariate Cox proportional hazards regression model for DFS.**

| **Variables** | **β Coefficient*** | **HR*** | **95% CI*** | ***P-*value*** | **Risk sore†** |
| --- | --- | --- | --- | --- | --- |
| **ICS** |  |  |  |  |  |
| Low risk | 1 |  |  |  | 0 |
| High risk | 1.927 | 6.871 | 3.023-15.616 | <0.001 | 1 |
| **TNM Stage** |  |  |  |  |  |
| I-III | 1 |  |  |  | 0 |
| IV | 1.309 | 3.701 | 1.528-8.961 | 0.004 | 1 |
| **NLR** |  |  |  |  |  |
| ≤ 4.5 | 1 |  |  |  | 0 |
| > 4.5 | 1.230 | 3.423 | 1.389-8.437 | 0.008 | 1 |

*Multivariate Cox proportional hazards regression model.

†Risk score was the rounded value obtained by dividing the β coefficient of each variable by β coefficient of TNM stage.

DFS, disease-free survival; ICS, inhibitory checkpoint-based signature; NLR, neutrophil-to-lymphocyte ratio; CI, confidence interval.
