# Supplementary figures and images for: Expression Profiles and Prognostic Value of Multiple Inhibitory Checkpoints in Head and Neck Lymphoepithelioma-Like Carcinoma
Source: Front Immunol. 2022 Jan 24;13:818411. doi: 10.3389/fimmu.2022.818411 (PMC8818848; doi:10.3389/fimmu.2022.818411)

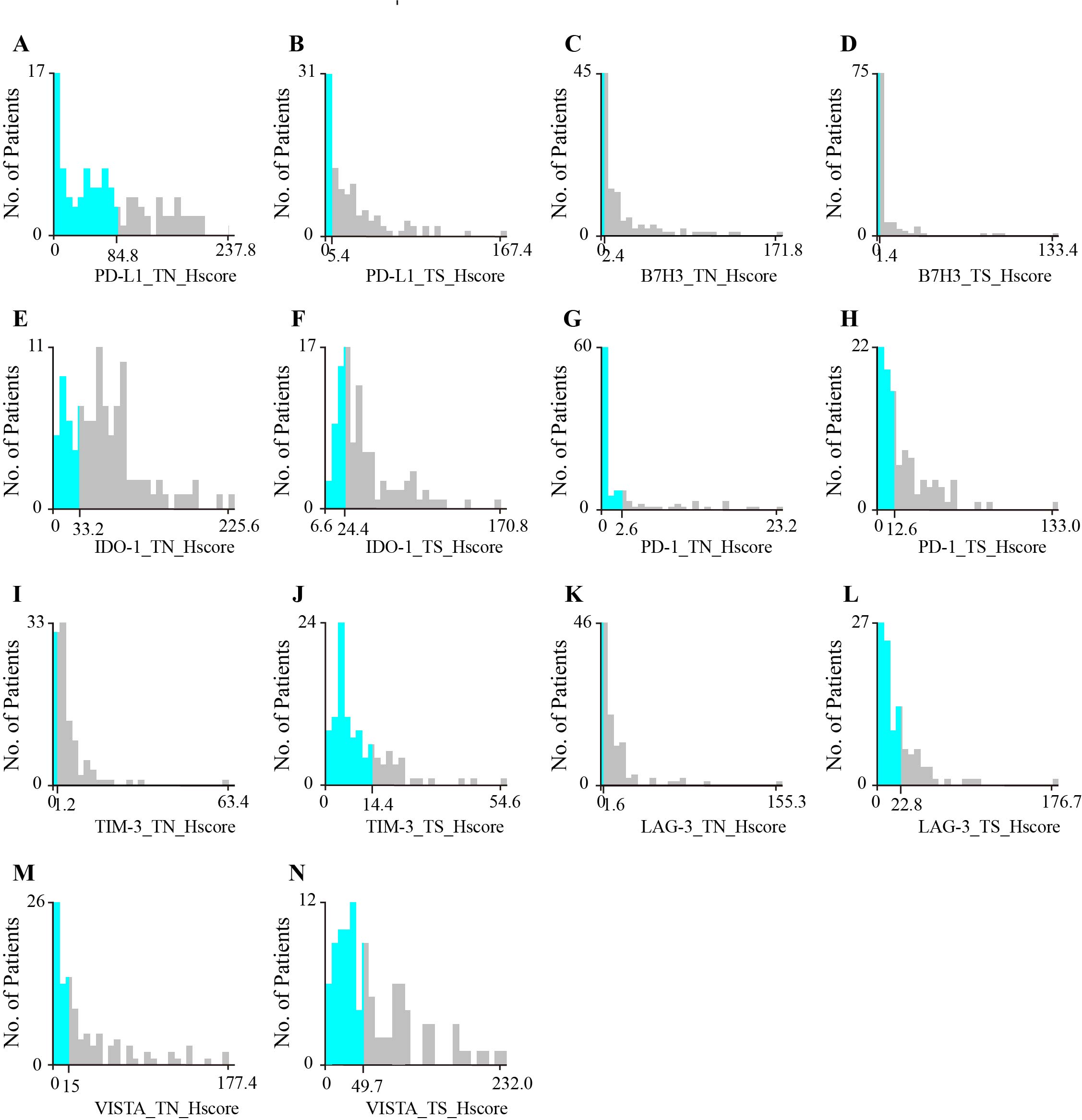

Supplement: Supplementary file 2 [file Image_1.jpg]

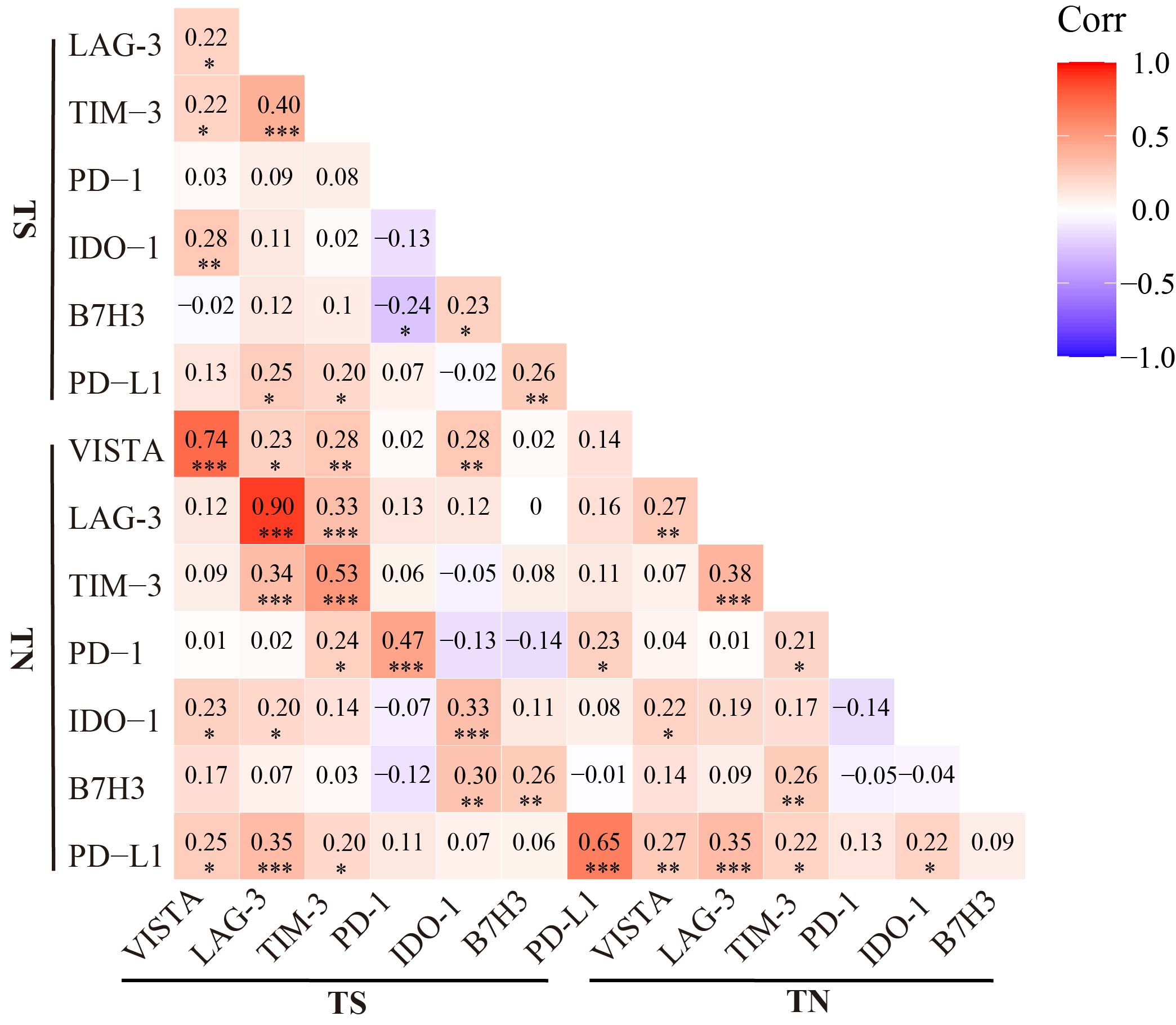

Supplement: Supplementary file 3 [file Image_2.jpg]

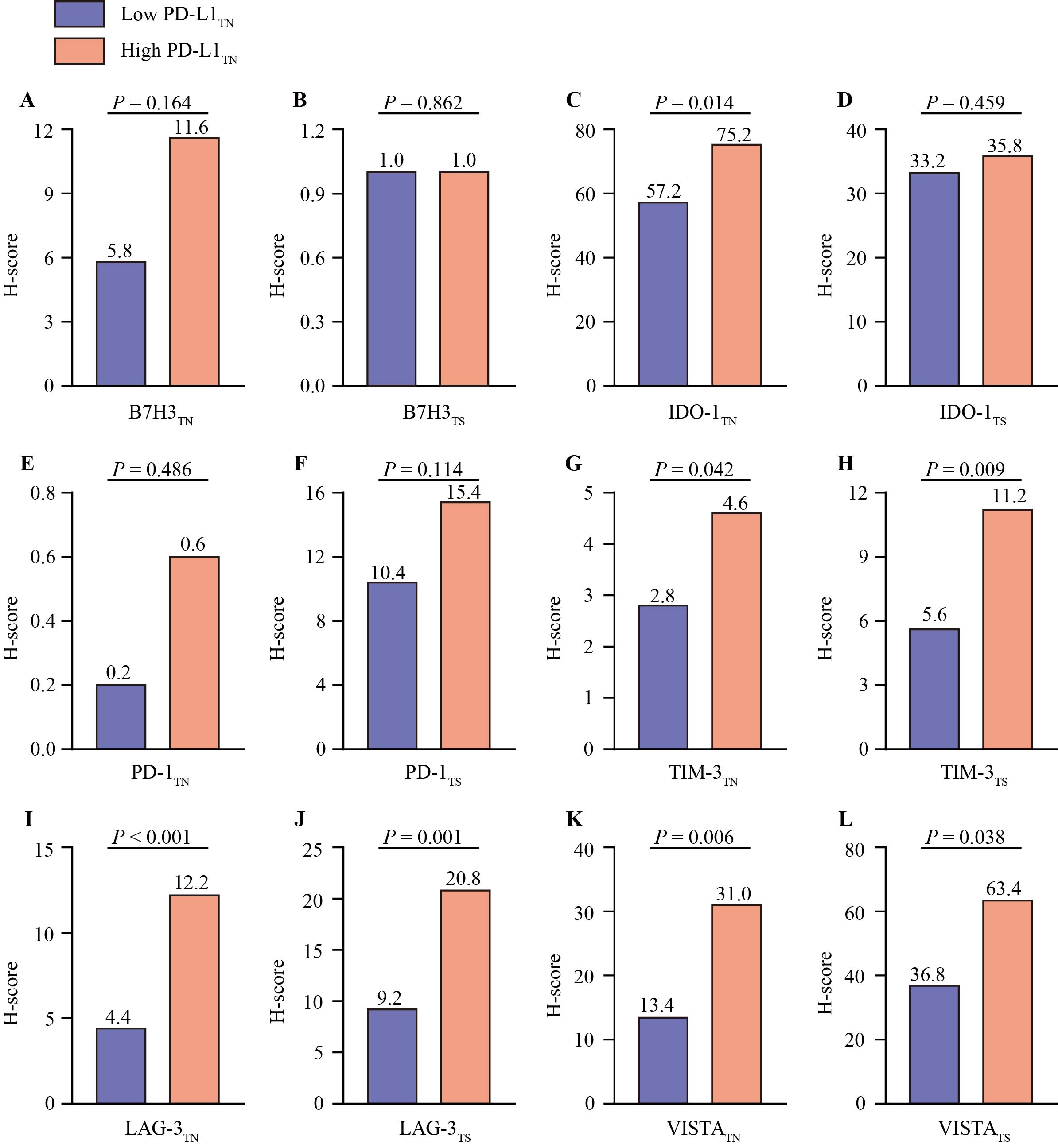

Supplement: Supplementary file 4 [file Image_3.jpg]

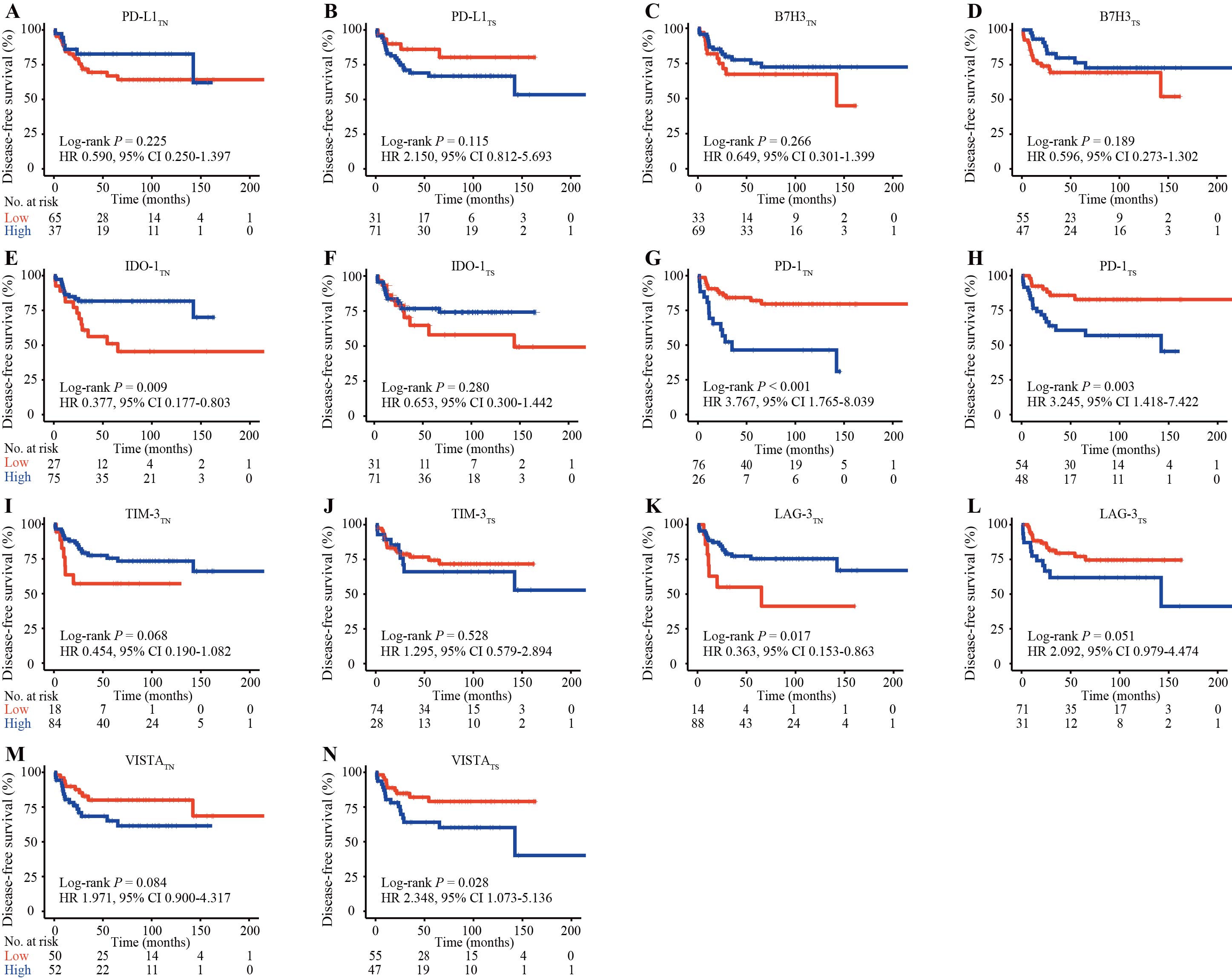

Supplement: Supplementary file 5 [file Image_4.jpg]

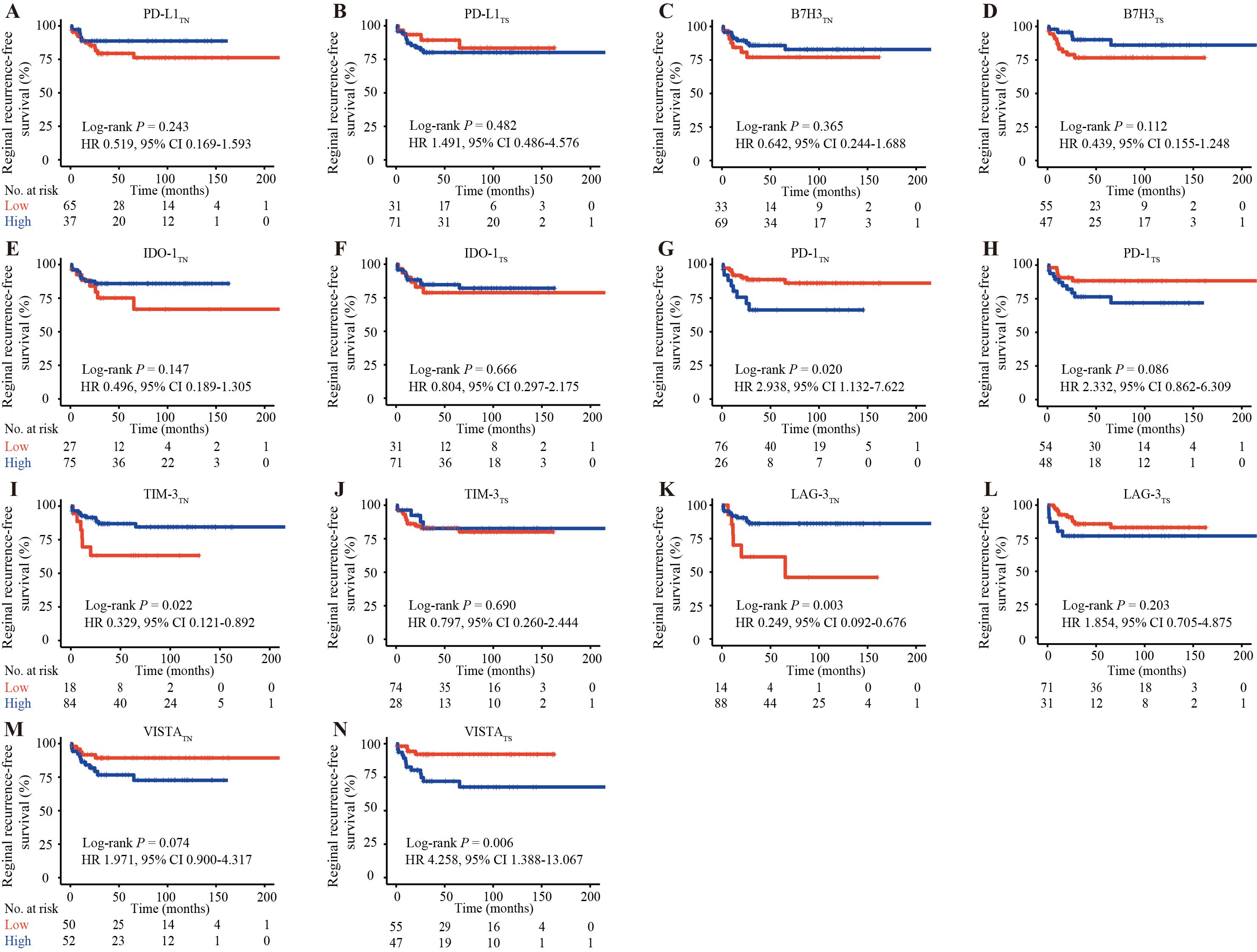

Supplement: Supplementary file 6 [file Image_5.jpg]

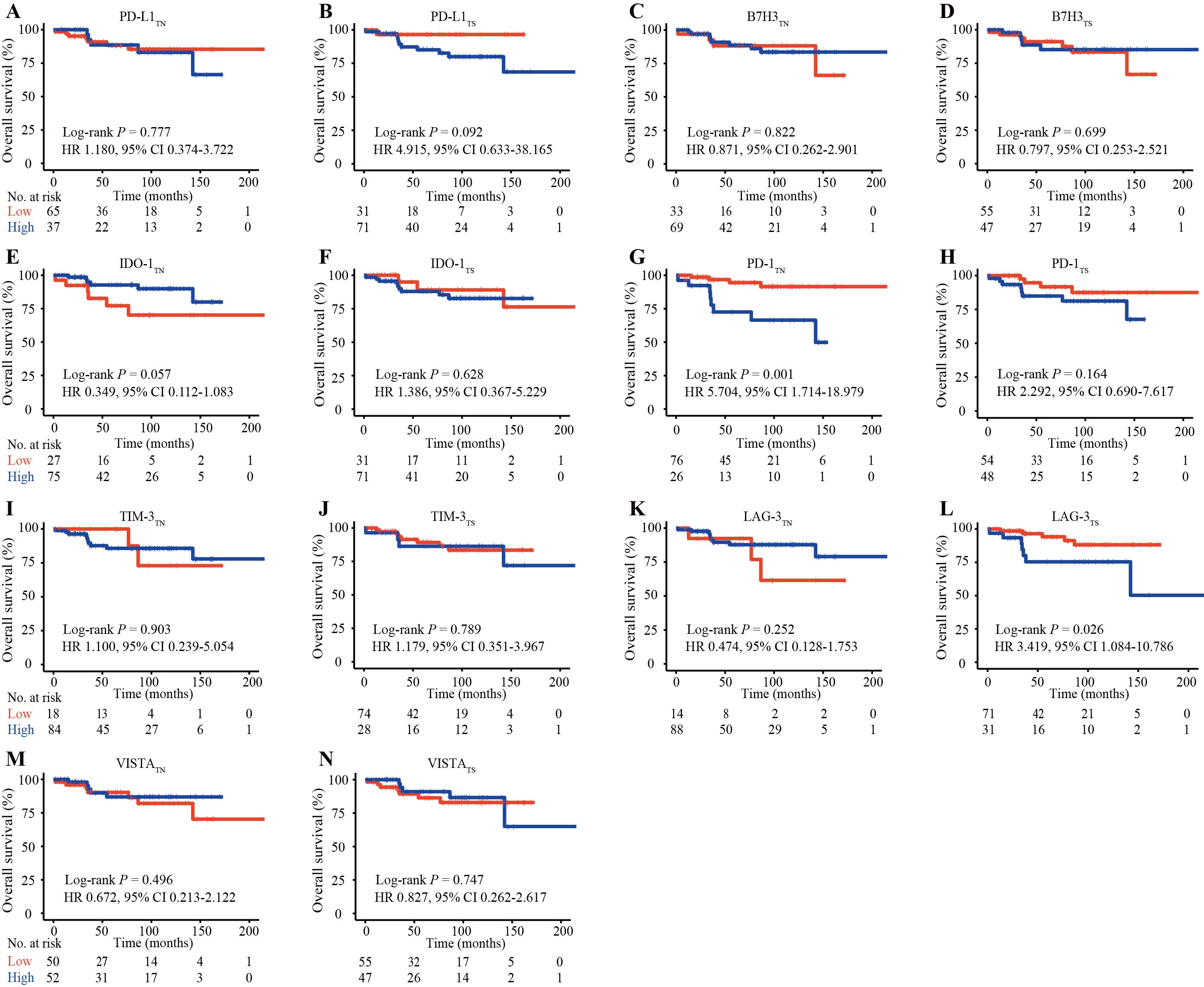

Supplement: Supplementary file 7 [file Image_6.jpg]

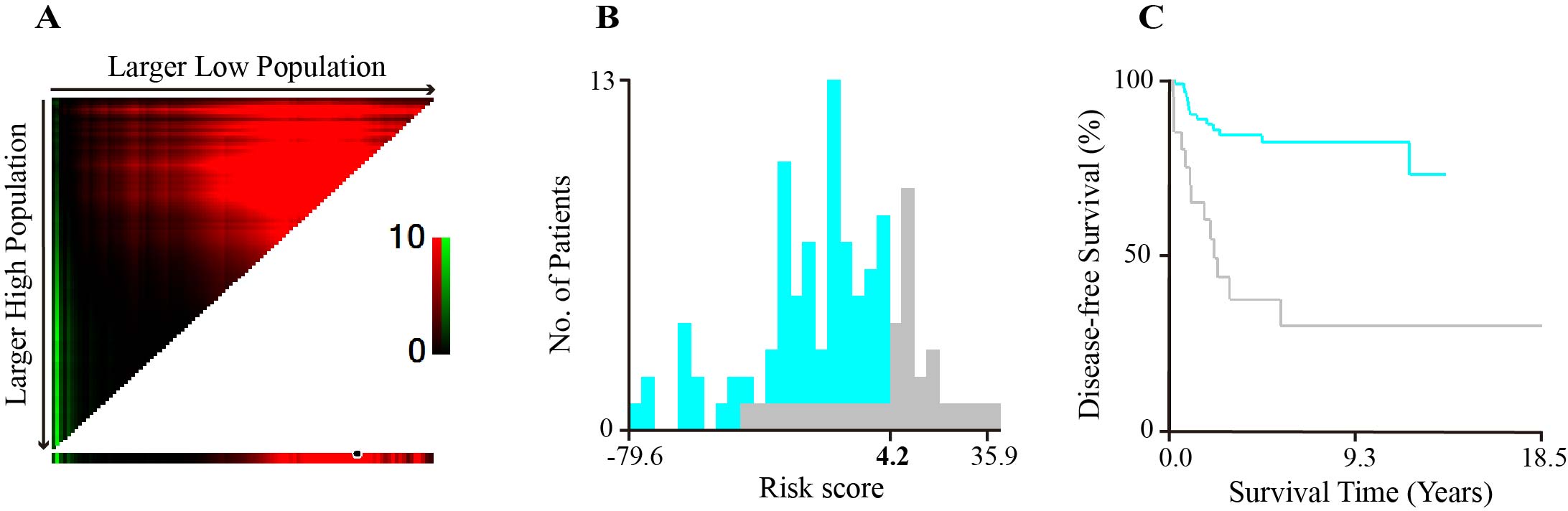

Supplement: Supplementary file 8 [file Image_7.jpg]

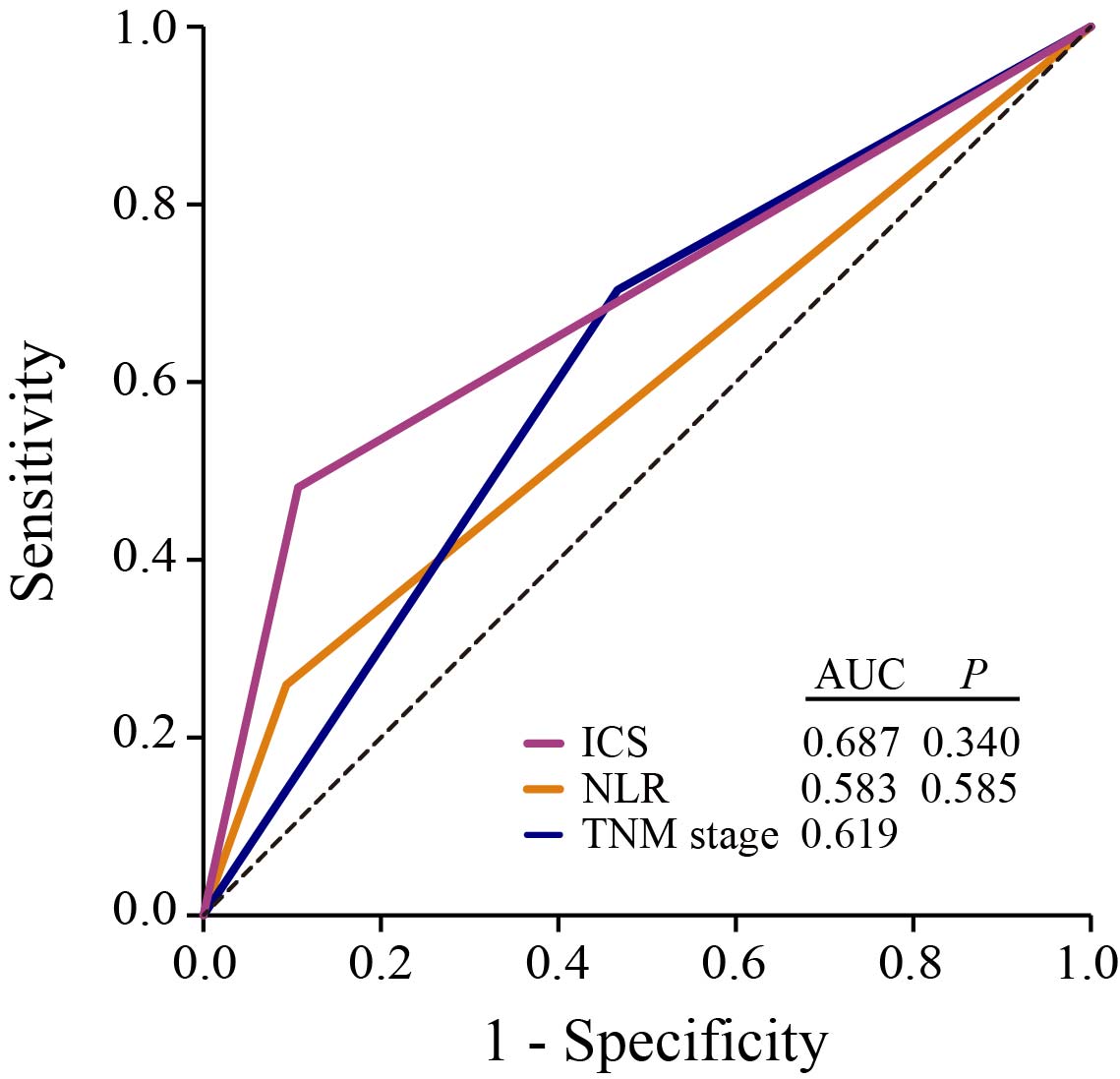

Supplement: Supplementary file 9 [file Image_8.jpg]
